# Germline Genetic Variants in *TEK*, *ANGPT1*, *ANGPT2*, *MMP9*, *FGF2* and *VEGFA* Are Associated with Pathologic Complete Response to Bevacizumab in Breast Cancer Patients

**DOI:** 10.1371/journal.pone.0168550

**Published:** 2017-01-03

**Authors:** Issam Makhoul, Valentina K. Todorova, Eric R. Siegel, Stephen W. Erickson, Ishwori Dhakal, Vinay R. Raj, Jeannette Y. Lee, Mohammed S. Orloff, Robert J. Griffin, Ronda S. Henry-Tillman, Suzanne Klimberg, Laura F. Hutchins, Susan A. Kadlubar

**Affiliations:** 1 Division of Hematology/Oncology Division, University of Arkansas for Medical Sciences, Little Rock, Arkansas, United States of America; 2 Division of Medical Genetics, University of Arkansas for Medical Sciences, Little Rock, Arkansas, United States of America; 3 Department of Biostatistics, University of Arkansas for Medical Sciences, Little Rock, Arkansas, United States of America; 4 Department of Epidemiology, University of Arkansas for Medical Sciences, Little Rock, Arkansas, United States of America; 5 Department of Radiation Oncology, University of Arkansas for Medical Sciences, Little Rock, Arkansas, United States of America; 6 Division of Breast Surgical Oncology, University of Arkansas for Medical Sciences, Little Rock, Arkansas, United States of America; 7 Department of Pathology, University of Arkansas for Medical Sciences, Little Rock, Arkansas, United States of America; Saint Louis University, UNITED STATES

## Abstract

**Background:**

We previously reported improved pathologic complete response (pCR) in a prospective phase II study using neoadjuvant bevacizumab in combination with chemotherapy compared to chemotherapy alone in breast cancer patients (41% vs. 25%, *p* = 0.0291). In this study, we queried germline single-nucleotide polymorphisms (SNPs) in angiogenesis-related genes for their impact on pCR and overall survival (OS).

**Methods:**

DNA for genotyping was available from 34 subjects who received bevacizumab in addition to chemotherapy and 29 subjects who did not. Using Illumina® technology, we queried 504 SNPs with a minor allele frequency (MAF) of at least 5%, located in 10 angiogenesis-related genes, for their effect on pCR via logistic regression with an additive-inheritance model while adjusting for race and bevacizumab treatment. SNPs that showed significant associations with pCR were selected for additional characterization.

**Results:**

After adjusting for race and tumor type, patients who had bevacizumab added to their neoadjuvant therapy were found to experience a significantly improved rate of pCR compared to patients who did not (adjusted OR 8.40, 95% CI 1.90–37.1). When patients were analyzed for SNP effects via logistic regression with race and bevacizumab treatment included as covariates, two SNPs in angiopoietin 1 (*ANGPT1)*, six in *ANGPT2*, three in fibroblast growth factor 2 (*FGF2)*, four in matrix metalloproteinase 9 (*MMP9*), three in tyrosine kinase, endothelial (*TEK*) and two in vascular endothelial growth factor A (*VEGFA*) were associated with pCR (P<0.05). However, when overall survival was considered, there was no difference between treatment groups or between genotypes.

**Conclusion:**

Genetic variability in *TEK*, *ANGPT1*, *ANGPT2*, *FGF2*, *MMP9 and VEGFA* is associated with pCR in bevacizumab-treated patients. Consistent with other studies, adding bevacizumab to standard chemotherapy did not impact OS, likely due to other factors and thus, while SNPs in *TEK*, *ANGPT1*, *ANGPT2*, *FGF2*, *MMP9 and VEGFA* were associated with pCR, they were not predictive of OS in this patient population.

**Trial Registration:**

ClinicalTrials.gov NCT00203502

## Introduction

Bevacizumab, a humanized monoclonal antibody that was designed to target vascular endothelial growth factor A (VEGF-A), was first approved for breast cancer in the metastatic setting in 2008 based on the results of the E2100 Intergroup phase III trial comparing paclitaxel with or without the addition of bevacizumab as first-line therapy. [1] The approval was contingent on the results of further studies, which ultimately did not demonstrate significant improvements in overall survival. Thus, FDA approval of bevacizumab for breast cancer was officially withdrawn in 2010 [2], although additional studies of bevacizumab in the adjuvant setting were ongoing. [3] In the neoadjuvant setting, the addition of bevacizumab to chemotherapy results in increased pathologic complete response [(pCR); reviewed in [4]]. While these results are promising, it is apparent that clinical response to bevacizumab is highly variable. For this reason, biomarkers are urgently needed to identify patients most likely to benefit.

Pharmacogenomics studies have reported associations between single nucleotide polymorphisms (SNPs) in *VEGF* and *VEGFR-2* and response in metastatic breast cancer [5–7], but studies of the impact of SNPs on pCR in the neoadjuvant setting have just started being performed. A recent analysis of the randomized phase III GeparQuinto study examined genetic variants within the VEGF pathway genes in relation to response to neoadjuvant bevacizumab. [8] In that study, a total of 15 genes were examined, but the only two common genes investigated in both that study and the current report were *KDR* and *VEGFA*. However, a comprehensive examination of germline SNPs in other genes involved in angiogenesis is lacking.

We have previously conducted and reported on a Phase II neoadjuvant trial examining the combination of chemotherapy with bevacizumab. [9] In that trial, we found significant differences in pCR according to tumor types (52% for ductal carcinoma compared to only 10% in non-ductal disease, p = 0.021), receptor status (59% in estrogen/progesterone receptor negative compared to only 27% in patients positive for at least one receptor, p = 0.047) and race (pCR in African Americans (AA) 75% compared to 28% in European Americans (EA), p = 0.007). Subsequent investigations in this patient population revealed that elevated serum levels of tyrosine kinase with immunoglobulin and epidermal growth factor-homology domains 2 (Tie-2) and basic fibroblast growth factor (bFGF) at baseline were seen in subjects who attained pCR. [10] In the current study, we combined two cohorts in order to assess the effects of the SNPs in the angiogenic pathway while adjusting for the effect of bevacizumab.

## Materials and Methods

### Study populations

Cancer patients–Data from two independent clinical studies were combined for the analyses in this report. One was a Phase II neoadjuvant clinical trial of bevacizumab added to chemotherapy, for which the study design, sample collection, and protein analysis have been described elsewhere. [9,10] The other was a prospective observational study investigating doxorubicin toxicity in breast-cancer patients who followed the same neoadjuvant chemotherapy regimen as the Phase II trial, but without the addition of bevacizumab; these patients were enrolled between 12/16/2010 and 9/1/2013. [11] Both studies were conducted at the University of Arkansas for Medical Sciences, and both had IRB approval. Serial blood samples were collected at baseline and at each visit from patients in both studies. From the Phase II trial, we excluded one Asian and one Hispanic to leave 37 patients who were either AA or EA. All 38 subjects from the observational study were AA or EA.

Healthy, cancer-free subjects–Healthy subjects were those recruited as controls in case-control studies of breast, colorectal and prostate cancer conducted at UAMS from 1997–2007. These studies were approved by the IRB at UAMS. All participants have signed a written consent form that was approved by the IRB of UAMS. Germline DNA was isolated from buffy coat samples provided by the healthy individuals.

### Genotyping assays

High-density genotyping–To test for the association between SNPs in candidate genes and pCR, DNA was extracted from buffy coats of 63 study participants (34 receiving bevacizumab/29 not). Genotyping was performed using the Illumina® Whole Genome Genotyping Infinium chemistry on both cohorts, with the HumanOmni2.5-8v1-1 BeadChip for the cohort receiving bevacizumab, and the HumanOmni5-4 BeadChip for the cohort not receiving bevacizumab. Individual samples with genotype call rates less than 99% and SNPs with call rates less than 95% were removed. The modest sample size precluded the ability to detect genome-wide associations, so even though many other genotypes were generated, we used a candidate gene/SNP approach for analysis.

Candidate SNP genotyping–Specific SNPs were selected for genotyping due to their degree of linkage with other SNPs in the gene under investigation. Genotype was determined using TaqMan^®^ SNP Genotyping Assays (Applied Biosystems) for rs2515462 and rs1960669 using 7900 HT Fast Real-Time PCR system (Applied Biosystems, Life Technologies, Carlsbad, California). Reactions were heated to 95°C for 10 minutes, and then subjected to 40 cycles of amplification at 95°C for 10 seconds and 60°C for 1 minute. PCR amplification was followed by allelic discrimination plate read and analysis.

### Statistical analysis

SNP Selection: SNPs were targeted for selection if they resided in one of 10 genes (*ANGPT1*, *ANGPT2*, *FGF2*, *IL1A*, *KDR*, *MMP9*, *PDGFB*, *PECAM1*, *TEK*, and *VEGFA*) that code for the proteins of our previous study. [10] This covered 833 SNPs on the HumanOmni2.5-8v1-1 BeadChip, and 1460 SNPs on the HumanOmni5-4 BeadChip system. SNPs were eligible for inclusion if they had matching SNP ID names plus matching chromosome + base positions on both chip systems. Differences in reported strand usage between cohorts were reconciled to match the so-called “dbsnp format–forward strand coding scheme” employed for the cohort that received bevacizumab. Eligible SNPs were included if they had a 100% call rate and an observed minor-allele frequency (MAF) >5% across all 63 genotyped samples; 504 SNPs qualified for inclusion into the initial genotype screen.

Statistical Analysis: Patient demographics and tumor characteristics were compared for differences between cohorts using the Wilcoxon rank-sum and chi-square tests. Prior to SNP assessment, the multivariate effect of cohort, race, and tumor type on pCR were evaluated via logistic regression, since race and tumor type showed significant associations with pCR in the Phase II trial of bevacizumab. [9] Because this evaluation showed that tumor type was not independently significant (*P* = 0.16) in the presence of cohort and race, it was ignored in the subsequent SNP assessments. For the initial genotype screen, the 504 SNPs were individually assessed for their effect on the odds of achieving pCR using a logistic-regression model with cohort and race included as covariates. The model assumed an additive mode of inheritance for the number of minor alleles carried by each subject. A SNP was selected for further analysis if the logistic regression returned an unadjusted P<0.05 for its effect on pCR, although its FDR-adjusted P-value was calculated for reporting purposes. Selected SNPs, in turn, had their minor-allele counts similarly assessed at P<0.05 via logistic regression for association with suitably dichotomized tumor characteristics (stage, grade, ER/PR status, HER2-Neu expression, triple-negative status, and histology). These logistic regressions included Race as an adjustment factor for every characteristic except histology because of the 100% prevalence of ductal carcinoma among African Americans in our two study cohorts. To analyze the selected SNPs for MAF imbalance between cohorts, subjects were subgrouped by race, and the MAF of each SNP was compared between cohorts within race via Fisher’s exact tests. Finally, for two of the 20 selected SNPs, the genotypes of both cohorts were combined and compared via chi-square tests for MAF differences with a race-matched cohort of healthy, cancer-free subjects.

## Results

### Study population demographics and characterization of pCR

**Fig 1:**
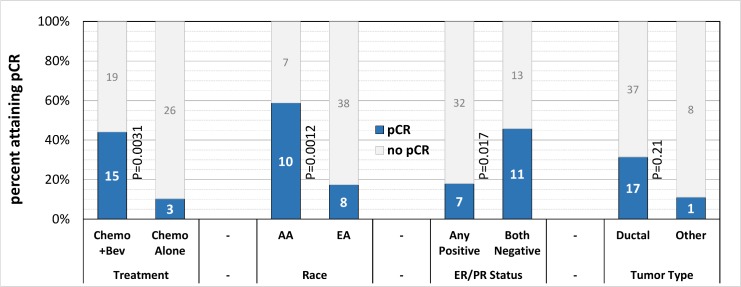
Rates of pCR by Treatment cohort, Race, Hormone-receptor status, and Tumor type among genotyped subjects. Stacked barchart showing how the number and proportion of pCRs varies by treatment cohort, by race, by ER/PR Status, and by tumor type. Group labels appear below their bars. P-values are from chi-square tests comparing groups for pCR differences.

The characteristics of the study population are detailed in Table 1. There were 37 patients who received additional bevacizumab with neoadjuvant chemotherapy, 33 of whom had genotype information available. Similarly, there were 38 patients who had standard neoadjuvant therapy, 29 of whom also had genotypes. The two groups differed significantly at P<0.05 with respect to race, age, tumor size, tumor type, HER2/Neu status, and lymph-node positivity (Table 1). Additionally, the number (%) of patients staged to a Stage III subgroup in Table 1 was 18 (49%) in the chemo + bevacizumab cohort compared to only 8 (21%) in the standard-chemotherapy cohort (P = 0.012). Table 1 demonstrates that patients in the bevacizumab cohort attained pCR much more frequently than patients in the standard chemo cohort. As shown in Fig 1, tumor-related characteristics associated with pCR among genotyped study subjects included hormone receptor negativity, but not ductal tumor type. Table 2 shows the multivariate effect of cohort, race, and tumor type on the odds of attaining pCR. The effect of cohort was independently significant (*P* = 0.0050), and the effect of race was marginally so (*P* = 0.051), but the effect of tumor type was insignificant (*P* = 0.16) in the presence of the other two covariates. As a consequence, tumor type was removed from the multivariate model; Table 3 shows the result. Both cohort and race had independently significant associations with pCR, and both were accordingly included as covariates to adjust for in the initial genotype screen.

**Table 1:** Patient demographics,tumor characteristics, and response to treatment.

*Patient demographic*:	Chemotherapy + Bevacizumab (N = 37)	Chemotherapy alone (N = 38)	P-value
**Race**, N (%)			0.046^†^
EA	25 (68%)	33 (87%)	
AA	12 (32%)	5 (13%)	
**Age (years)**			0.013^‡^
Median	43	51	
quartiles	37–55	44–61	
Range	26–72	35–76	
**BMI (kg/m**^**2**^**)**			0.23^‡^
Median	26	29	
quartiles	24–30	24–32	
Range	17–47	19–41	
*Tumor Characteristic*:			
**Tumor size (cm)**			0.0057^‡^
Median	5.5	4.0	
quartiles	4.0–7.0	3.0–5.5	
Range	2.0–15.0	1.3–25.0	
**Tumor Type**, N (%)			0.0057^†^
Ductal	28 (76%)	37 (97%)	
Non-Ductal^1^	9 (24%)	1 (3%)	
**Grade**, N (%)			0.83 ^‡^
I	2 (5%)	3 (8%)	
II	12 (32%)	12 (32%)	
III	23 (62%)	23 (61%)	
**TNM Stage**, N (%)			0.012^†^^4^
I^2^	0 (0%)	1 (3%)	
IIA^2^	7 (19%)	15 (39%)	
IIB^2^	12 (32%)	14 (37%)	
IIIA^3^	16 (43%)	7 (18%)	
IIIB^3^	2 (5%)	0 (0%)	
IIIC^3^	0 (0%)	1 (3%)	
**Hormone Receptor status**, N (%):			0.13^†^
ER+ or PR+	20 (54%)	27 (71%)	
ER-/PR-	17 (46%)	11 (29%)	
**HER-2/Neu Status**, N(%)			0.037^†^
HER2+	8 (22%)	2 (5%)	
HER2–	29 (78%)	36 (95%)	
**Triple negativity**, N (%)			0.41^†^
Yes	14 (38%)	11 (29%)	
No	23 (62%)	27 (71%)	
**Lymph-node status**, N (%)			0.012^†^
Positive	28 (76%)	18 (47%)	
Negative	9 (24%)	20 (53%)	
*Response to Treatment*:			
**pCR**^**5**^, N (%)			0.0037^†^
Yes	16 (43%)	5 (13%)	
No	21 (57%)	33 (87%)	

P-values are via the

†chi-square test or

‡Wilcoxon rank-sum test.

1: Non-ductal tumor types included 6 lobular and 3 poorly differentiated in the Chemotherapy + Bevacizumab cohort, and one metaplastic in the Chemotherapy Alone cohort.

2: Early TNM stage.

3: Late TNM stage.

4: The chi-square test compared early TNM stages to late TNM stages.

5: Pathologic Complete Response.

**Table 2:** Multivariate evaluation of cohort, race, and tumor type on pCR.

Effect	Adjusted Odds Ratio	95% Confidence Interval	*P*
**Cohort:**			
Bevacizumab	8.40	1.90–37.1	0.0050
Standard chemotherapy^†^	1.00	–––––	
**Race:**			
African American	4.05	0.99–16.6	0.051
European American^†^	1.00	–––––	
**Tumor Type:**			
Ductal carcinoma	5.37	0.53–54.8	0.16
Other histology^†^	1.00	–––––	

† Reference category

**Table 3:** Effect of cohort and race on pCR after removing tumor type.

Effect	Adjusted Odds Ratio	95% Confidence Interval	*P*
**Cohort:**			
Bevacizumab	6.03	1.41–25.7	0.015
Standard chemotherapy^†^	1.00	–––––	
**Race:**			
African American	5.96	1.59–22.3	0.0081
European American^†^	1.00	–––––	

† Reference category

### Initial genotyping screen

Twenty of the 504 SNPs assessed in the initial genotype screen yielded unadjusted P-values <0.05. They included two SNPs in *ANGPT1*, six in *ANGPT2*, three in *FGF2*, four in *MMP9*, three in *TEK* and two in *VEGFA* (Table 4). Although none of the 20 SNPs retained significance after adjusting for multiple comparisons (FDR-adjusted P = 0.88), they were selected for further analysis. Tables 5 and 6 show the minor-allele frequencies of the 20 selected SNPs in the two breast-cancer cohorts receiving neoadjuvant chemotherapy. There were no significant differences in the distribution of alleles between the Bevacizumab cohort and the chemotherapy-alone cohort among AA subjects (Table 5), but significant differences in *FGF2* and *TEK* allele frequencies were evident in EA subjects (Table 6). However, there was no significant difference in pCR for EA patients between the two cohorts (6 pCR /22 EA with bevacizumab versus 2 pCR /24 EA with chemotherapy alone; Fisher’s exact *P* = 0.13), suggesting that differences in allele frequency were due to chance, given the small number of subjects. None of the other 16 SNPs were significantly different between cohorts in EA subjects. We also examined these 20 SNPs in relation to tumor characteristics (Table 7). One SNP in *TEK* (kgp3874749) was negatively associated with higher stage of disease while another SNP in *FGF2* (kgp11559855) was positively associated with both double-negative ER/PR status and triple-negative ER/PR/HER2-Neu status. Two closely linked SNPs in *VEGFA* were positively associated with ductal carcinoma. No SNP was associated with Grade or with HER2-Neu expression as a single factor.

**Table 4:** Odds ratios (95% CI) for pCR by Genotype.

Gene	Chr	SNP ID	pCR?	Minor-allele copies			*AOR	**Raw P
				0	1	2	(95% CI)	
*ANGPT1*	8	kgp364928	No	23	18	4	0.26 (0.07–0.99)	0.049
			Yes	14	3	1		
*ANGPT1*	8	rs2445365	No	30	15	0	6.75 (1.41–32.4)	0.017
			Yes	8	10	0		
*ANGPT2*	8	rs2515409	No	33	11	1	0.12 (0.02–0.84)	0.032
			Yes	15	3	0		
*ANGPT2*	8	rs10102851	No	41	4	0	6.14 (1.09–34.7)	0.04
			Yes	9	7	2		
*ANGPT2*	8	rs2515462	No	26	18	1	4.31 (1.40–13.3)	0.011
			Yes	6	7	5		
*ANGPT2*	8	kgp2706843	No	18	21	6	0.27 (0.08–0.92)	0.036
			Yes	11	6	1		
*ANGPT2*	8	rs13269021	No	20	23	2	0.24 (0.06–0.96)	0.044
			Yes	12	6	0		
*ANGPT2*	8	rs1375668	No	22	20	3	2.98 (1.01–8.83)	0.049
			Yes	3	9	6		
*FGF2*	4	kgp6880528	No	41	4	0	8.54 (1.09–66.75)	0.041
			Yes	14	4	0		
*FGF2*	4	kgp11559855	No	37	7	1	0.16 (0.03–0.99)	0.049
			Yes	15	3	0		
*FGF2*	4	rs1960669	No	33	11	1	0.12 (0.02–0.79)	0.027
			Yes	15	3	0		
*MMP9*	20	rs2274755	No	37	8	0	8.97 (1.44–56.0)	0.019
			Yes	11	7	0		
*MMP9*	20	rs2236416	No	37	8	0	18.7 (1.97–178.1)	0.011
			Yes	10	8	0		
*MMP9*	20	rs2274756	No	37	8	0	8.97 (1.44–56.0)	0.019
			Yes	11	7	0		
*MMP9*	20	kgp5474569	No	37	8	0	8.97 (1.44–56.0)	0.019
			Yes	11	7	0		
*TEK*	9	kgp2288251	No	37	6	2	4.45 (1.21–16.4)	0.025
			Yes	11	6	1		
*TEK*	9	kgp4866617	No	38	5	2	6.88 (1.53–30.9)	0.012
			Yes	13	4	1		
*TEK*	9	kgp3874749	No	17	26	2	3.47 (1.10–10.9)	0.033
			Yes	5	7	6		
*VEGFA*	6	rs833068	No	26	16	3	3.67 (1.16–11.60)	0.027
			Yes	3	11	4		
*VEGFA*	6	rs833069	No	25	17	3	4.95 (1.37–17.9)	0.015
			Yes	2	11	5		

*AOR (95% CI): Adjusted odds ratio for pCR with a 1-unit increase in the number of minor alleles adjusted for race and bevacizumab, 95% confidence interval.

** Unadjusted P-value associated with the AOR.

The FDR-adjusted P-value for all 20 SNPs was 0.88

**Table 5:** SNP allele distribution by cohort in African American (AA) subjects.

Gene	SNP ID	Minor allele	Major allele	*MAF (C+Bev)	*MAF (Chemo)	Fisher's Exact P-value
*ANGPT1*	kgp364928	C	T	0.2917	0.3000	1.0000
*ANGPT1*	rs2445365	T	C	0.1667	0.1000	1.0000
*ANGPT2*	rs2515409	C	T	0.2500	0.3000	1.0000
*ANGPT2*	rs10102851	G	A	0.4167	0.2000	0.4322
*ANGPT2*	rs2515462	A	G	0.3750	0.3000	1.0000
*ANGPT2*	kgp2706843	A	G	0.3750	0.5000	0.7041
*ANGPT2*	rs13269021	T	G	0.2500	0.1000	0.6445
*ANGPT2*	rs1375668	G	A	0.6250	0.7000	1.0000
*FGF2*	kgp6880528	A	G	0.0417	0.1000	0.5080
*FGF2*	kgp11559855	G	T	0.2500	0.2000	1.0000
*FGF2*	rs1960669	T	G	0.2083	0.1000	0.6445
*MMP9*	rs2274755	T	G	0.1250	0.1000	1.0000
*MMP9*	rs2236416	G	A	0.1250	0.2000	0.6181
*MMP9*	rs2274756	A	G	0.1250	0.1000	1.0000
*MMP9*	kgp5474569	A	G	0.1250	0.1000	1.0000
*TEK*	kgp2288251	G	A	0.2083	0.0000	0.2908
*TEK*	kgp4866617	A	T	0.0833	0.0000	1.0000
*TEK*	kgp3874749	C	T	0.5000	0.6000	0.7146
*VEGFA*	rs833068	A	G	0.5833	0.4000	0.4569
*VEGFA*	rs833069	G	A	0.6667	0.5000	0.4505

* Minor Allele Frequency by cohort, calculated for 12 AA in the bevacizumab (C+Bev) cohort versus 5 AA in the standard-chemotherapy (Chemo) cohort.

**Table 6:** SNP Allele Distribution by cohort in European American (EA) subjects.

Gene	SNP ID	Minor allele	Major allele	*MAF (C+Bev)	*MAF (Chemo)	Fisher's Exact P-value
*ANGPT1*	kgp364928	C	T	0.1818	0.2708	0.3328
*ANGPT1*	rs2445365	T	C	0.1818	0.2500	0.4597
*ANGPT2*	rs2515409	C	T	0.0909	0.0625	0.7058
*ANGPT2*	rs10102851	G	A	0.0227	0.0417	1.0000
*ANGPT2*	rs2515462	A	G	0.2500	0.2917	0.8149
*ANGPT2*	kgp2706843	A	G	0.2955	0.2917	1.0000
*ANGPT2*	rs13269021	T	G	0.3182	0.2500	0.4957
*ANGPT2*	rs1375668	G	A	0.2727	0.2708	1.0000
*FGF2*	kgp6880528	A	G	0.0455	0.0833	0.6788
*FGF2*	kgp11559855	G	T	0.0909	0.0000	**0.0486**
*FGF2*	rs1960669	T	G	0.2045	0.0208	**0.0060**
*MMP9*	rs2274755	T	G	0.0682	0.1667	0.2025
*MMP9*	rs2236416	G	A	0.0682	0.1667	0.2025
*MMP9*	rs2274756	A	G	0.0682	0.1667	0.2025
*MMP9*	kgp5474569	A	G	0.0682	0.1667	0.2025
*TEK*	kgp2288251	G	A	0.0227	0.2500	**0.0019**
*TEK*	kgp4866617	A	T	0.0227	0.2500	**0.0019**
*TEK*	kgp3874749	C	T	0.2500	0.4167	0.1227
*VEGFA*	rs833068	A	G	0.1818	0.3125	0.2278
*VEGFA*	rs833069	G	A	0.1818	0.3125	0.2278

* Minor Allele Frequency by cohort, calculated for 22 EA in the bevacizumab (C+Bev) cohort versus 24 EA in the standard-chemotherapy (Chemo) cohort.

**Table 7:** SNPs associated with patient characteristics.

Outcome	Event (vs non-event)	Gene	SNP ID	Estimated log(OR)^1^	Standard error	P-value
AJCC Stage	IIIA/B/C (vs IIA/B)^2^	TEK	kgp3874749	-1.027	0.469	0.028
ER/PR^3^ status	Both Negative (vs Any Positive)	FGF2	kgp11559855	1.606	0.759	0.034
ER/PR/HER2-Neu status	All Three Negative (vs Any Positive)	FGF2	kgp11559855	1.783	0.769	0.020
Histology	Ductal (vs Other)	VEGFA	rs833068	2.349^†^	1.068	0.028
Histology	Ductal (vs Other)	VEGFA	rs833069	2.485^†^	1.071	0.020

1: Estimated natural log of the Odds Ratio (OR) favoring the event over the non-event with each unit increase in the subject’s minor-allele count, as determined by logistic regression. Estimates are adjusted for Race for all outcomes except †Histology, where the 100% prevalence of ductal carcinoma among African Americans precluded a Race adjustment.

2: See **Table 1** for the numeric breakdown of AJCC stages.

3: Estrogen Receptor / Progesterone Receptor.

### Allele frequency distribution between cancer patients and healthy subjects

Response to Bevacizumab in the clinical trial varied significantly by race. Thus, we examined differences in allele frequency by race (data not shown). We also selected two representative SNPs to examine whether they displayed differences in allele frequencies between the breast cancer patients and healthy individuals from the same population. There were no differences in allele frequencies compared to healthy individuals, suggesting that the two selected SNPs are involved with treatment response and are not risk alleles for breast cancer (Tables 8 and 9**)**.

**Table 8:** Comparison in European Americans (EA) of allele frequencies between cancer patients and healthy population.

Gene (SNP)	Cancer Patients (N = 46 EA subjects)	Healthy Population (N = 45 EA subjects)	P^1^
*ANGPT2* (rs2515462)	G: 73% (67/92)	G: 71% (64/90)	0.80
	A: 27% (25/92)	A: 29% (26/90)	
*FGF2* (rs1960669)	G: 89% (82/92)	G: 89% (80/90)	0.96
	T: 11% (10/92)	T: 11% (10/90)	

1: P-values are from chi-square tests.

**Table 9:** Comparison in African Americans (AA) of allele frequencies between cancer patients and healthy population.

Gene (SNP)	Cancer Patients (N = 17 AA subjects)	Healthy Population (N = 39 AA subjects)	P^1^
*ANGPT2* (rs2515462)	G: 65% (22/34)	G: 54% (42/78)	0.29
	A: 35% (12/34)	A: 46% (36/78)	
*FGF2* (rs1960669)	G: 82% (28/34)	G: 87% (68/78)	0.50
	T: 18% (6/34)	T: 13% (10/78)	

1: P-values are from chi-square tests.

## Discussion

Since the introduction of anti-angiogenic therapy targeting VEGF to clinical practice in the late 1990s-early 2000s, noted differences in efficacy have been observed between cancer types and between patients within the same cancer type. The efficacy of bevacizumab as an anti-angiogenic agent for breast cancer has generated mixed findings. A recent meta-analysis, however, reports that in the neoadjuvant setting, the addition of Bev to standard chemotherapy significantly improves pCR, particularly in patients who are HER2-negative and hormone receptor-negative. [12] While these tumor characteristics are good predictors of pCR, the incorporation of other variables, such as germline SNPs in the angiogenic pathway, could potentially improve prediction models.

Our overarching hypothesis was that the interaction of the type of cancer and the host’s angiogenic profile explains differences in clinical response to VEGF inhibition. In order for a cancer to be sensitive to the interruption of angiogenesis, it has to be undergoing active blood vessel remodeling at the time of treatment. The maturation of blood vessels in many cancers is so advanced by the time of the diagnosis that targeting the machinery for angiogenesis may, at best, lead to stabilization of the tumor rather than its eradication. Clearly, tumor characteristics associated with response to the addition of bevacizumab to chemotherapy are hormone-receptor negativity and ductal type. These cancers are usually highly proliferative, high-grade cancers that are responsive to chemotherapy. Defining the host’s angiogenic profile is more problematic. The last four decades of angiogenesis research have unraveled the multiplicity and redundancy of pathways involved in this crucial process for multicellular organisms. [13] Hence, targeting one pathway when the host may offer the tumor alternative pathways to rescue angiogenesis is not likely to succeed. Alternatively, success is more likely if the targeted pathway is the only one–or the main one–for a specific host.

Analysis of angiogenesis-related proteins in our patients’ serum at baseline showed that high serum Tie2 and high serum bFGF were associated with pCR. [10] Baseline levels of serum ANG1 and ANG2 and their ratios did not show a significant association with pCR. Their levels remained stable during treatment regardless of the response, which suggested that these proteins were related to the host and not the tumor. [10]

In this study, we found that specific SNPs in some of the host angiogenic genes were associated with pCR in the neoadjuvant setting in breast cancer patients receiving bevacizumab and chemotherapy. In our previous study, some of the proteins encoded by these genes were elevated in patients who achieved pCR after receiving bevacizumab; this was the case for bFGF and Tie2, proteins encoded by *bFGF* and *TEK* genes, respectively. However, it is interesting that some of these proteins (Tie2) and genes (*TEK*, *ANGP1* and *2*) are components of the same pathway. Other pathways may also be involved, including *MMP9* and *VEGFA*. Results from the randomized phase III GeparQuinto study identified five SNPs in the host VEGF-pathway genes that were associated with improved pCR, particularly in patients with triple negative disease. [8] However, there was no overlap in the 15 genes tested in that study with variants investigated in our study except for the *KDR* and *VEGFA* genes. The strongest association with pCR in the GeparQuinto study was reported for rs833058, which is located upstream of the *VEGFA* promoter, whereas in our study, rs833068 and rs833069 were significantly associated with pCR. These SNPs are located within approximately 10,000bp of each other and are likely to be linked. Taken together, these reports indicate that genetic variation in *VEGFA* can influence response to neoadjuvant bevacizumab. This difference between our study and the GeparQuinto study could be due to differences in patient populations and to the size of the studies. Additionally, the GeparQuinto study reported interactions between *VEGFA* SNPs and hormone receptor status, while our study indicated associations between *VEGFA* SNPs and ductal carcinoma, and *FGF2* SNPs and receptor status. Again, these differences are likely due to sample sizes and population differences.

Results from the two studies combined for analysis in this report indicate that pCR is improved with the addition of bevacizumab. When overall survival was examined, there was no difference between the cohorts of our study. While this may appear as a shortcoming for this work, in reality, it is likely due to the fact that survival was extremely high (>95%) in both groups (data not shown). What is intriguing in our study is the observation that AA women had much improved pCR compared to women of European ancestry. Whether this finding could be due to a race-related difference in the tumor’s responsiveness to bevacizumab is deserving of further study to fully evaluate if *ANGPT1*, *ANGPT2*, *TEK*, *FGF2*, *MMP9* and *VEGFA* are promising targets for future research. Genotyping analysis results suggest that the interaction of the host’s angiogenic profile and the type of cancer may contribute to differences in clinical response to VEGF inhibition. Therefore, it is possible that variants of the *ANGPT1*, *ANGPT2*, *TEK*, *FGF2*, *MMP9* and *VEGFA* genes or their respective proteins might render certain tumors more susceptible to targeting of VEGF. These findings are hypothesis-generating and should be validated in a larger ethnically diverse population. The authors have initiated the process to access DNA from SWOG S0800, a randomized open-label phase II clinical trial that compared the combination of weekly nab-paclitaxel and bevacizumab followed by dose-dense doxorubicin and cyclophosphamide (AC) with nab-paclitaxel followed or preceded by AC as neoadjuvant treatment for HER2-negative locally advanced breast cancer. The primary endpoint was pCR. A total of 215 patients were accrued including 32% with TNBC. The addition of bevacizumab significantly increased the pCR rate overall (36 vs. 21%; p = 0.019) and in TNBC (59 vs. 29%; p = 0.014), but not in hormone receptor-positive disease (24 vs. 18%; p = 0.41). [14] Baseline and on-treatment blood samples were collected. The results of this trial are consistent with our findings and make it a perfect setting to validate our hypothesis.

## Supporting Information

S1 File Dictionary. **AngioSNPs data dictionary.** This Excel file contains the Data Dictionary for the ten other Excel files included as Supporting Information; they are described below.
(XLSX)

S2 File Clinical. **Clinical Data for Both Cohorts.** This Excel file contains the clinical information on the 63 genotyped cohorts in the study. It includes each subject’s cohort, race, tumor type, receptor status, and whether or not she attained pCR.
(XLSX)

S3 File Bev Samples. **BEV Ten-gene Sample Genotypes.** This Excel file contains the genotype information for the 34 subjects in the Bevacuzumab cohort. The genotype information consists of the alleles determined for every SNP inside the 10 target genes that were included on Illumina’s HumanOmni2.5-8v1-1 BeadChip.
(XLSX)

S4 File Bev Anno. **BEV Ten-gene SNP Annotations.** This Excel file contains the SNP annotations for all SNPS inside the 10 target genes that were included on Illumina’s HumanOmni2.5-8v1-1 BeadChip.
(XLSX)

S5 File Dox Samples. **DOX Ten-gene Sample Genotypes.** This Excel file contains the genotype information for the 29 subjects in the Doxorubicin cohort. The genotype information consists of the alleles determined for every SNP inside the 10 target genes that were included on Illumina’s HumanOmni5-4 BeadChip.
(XLSX)

S6 File Dox Anno. **DOX Ten-gene SNP Annotations.** This Excel file contains the SNP annotations for all SNPS inside the 10 target genes that were included on Illumina’s HumanOmni5-4 BeadChip for all SNPS inside the 10 target genes.
(XLSX)

S7 File Dox Strand. **DOX Ten-gene SNP Strand Info.** This Excel file contains the SNP strand-usage information for the genotype data in the Doxorubicin cohort (S5 File). It is needed to reconcile the strand usages in the Doxorubicin cohort with those in the Bevacizumab cohort after combining the genotype data for both cohorts.
(XLSX)

S8 File Reconciled. **Both Cohorts Reconciled Strands.** This Excel file contains the results after combining the genotype data from the two cohorts and making strand usage in the Doxorubicin cohort match that in the Bevacizumab cohort at any SNP where strand usage originally differed between cohorts. These results were used as inputs to generate the data shown in the S9, S10, and S11 Files.
(XLSX)

S9 File AlleleFreqs. **Allele Frequencies of All SNPs.** This Excel file contains the observed allele frequencies of all SNPs of interest in the two cohorts combined. The results in this file were used to filter the SNPs, i.e., to exclude from further analysis any SNP whose observed minor-allele frequency was less than 5% among the 63 subjects in the analysis.
(XLSX)

S10 File FinalSNPs. **Both Cohorts Final SNPs (bases).** This Excel file contains the genotype data (i.e., the 126 base calls per SNP) for the SNPs whose observed minor-allele frequencies were greater than 5% among the 63 subjects in the analysis.
(XLSX)

S11 File MinorCounts. **Minor-Allele Counts per subject.** This Excel file contains, for each SNP in the analysis, the number of minor alleles carried by each subject in the analysis. This file and S2 File Clinical together contain the data used in the logistic regressions.
(XLSX)

S12 File Logistic Clinical. ** Logistic Regressions vs Cohort, Race, and Tumor Chars.** This Word document contains the results of logistic regression of pCR on Cohort, Race, and each of several tumor characteristics taken one at a time, using the 63 subjects who had been genotyped. No SNPs were included.
(DOCX)
