# Supplementary material for: Germline Genetic Variants in TEK, ANGPT1, ANGPT2, MMP9, FGF2 and VEGFA Are Associated with Pathologic Complete Response to Bevacizumab in Breast Cancer Patients
Source: PLoS One. 2017 Jan 3;12(1):e0168550. doi: 10.1371/journal.pone.0168550 (PMC5207665; doi:10.1371/journal.pone.0168550)
Supplement: S12 File — Logistic Regressions vs Cohort, Race, and Tumor Chars. This Word document contains the results of logistic regression of pCR on Cohort, Race, and each of several tumor characteristics taken one at a time, using the 63 subjects who had been genotyped. No SNPs were included. (DOCX) [file pone.0168550.s012.docx]

| **Number of Observations Read** | 63 |
| --- | --- |
| **Number of Observations Used** | 63 |

| **Response Profile** | | |
| --- | --- | --- |
| **Ordered Value** | **pCR** | **Total Frequency** |
| **1** | no | 45 |
| **2** | yes | 18 |

| **Probability modeled is pCR='yes'.** |
| --- |

| **Type 3 Analysis of Effects** | | | |
| --- | --- | --- | --- |
| **Effect** | **DF** | **Wald Chi-Square** | **Pr > ChiSq** |
| **Cohort** | 1 | 5.8921 | 0.0152 |
| **RACE** | 1 | 7.0084 | 0.0081 |

| **Analysis of Maximum Likelihood Estimates** | | | | | | |
| --- | --- | --- | --- | --- | --- | --- |
| **Parameter** |  | **DF** | **Estimate** | **Standard Error** | **Wald Chi-Square** | **Pr > ChiSq** |
| **Intercept** |  | 1 | -2.6760 | 0.6946 | 14.8444 | 0.0001 |
| **Cohort** | **Bevacizumab** | 1 | 1.7970 | 0.7403 | 5.8921 | 0.0152 |
| **RACE** | **B** | 1 | 1.7848 | 0.6742 | 7.0084 | 0.0081 |

| **Odds Ratio Estimates** | | | |
| --- | --- | --- | --- |
| **Effect** | **Point Estimate** | **95% Wald Confidence Limits** | |
| **Cohort Bevacizumab vs Doxorubicin** | 6.031 | 1.413 | 25.738 |
| **RACE B vs W** | 5.959 | 1.590 | 22.337 |

| **Number of Observations Read** | 63 |
| --- | --- |
| **Number of Observations Used** | 63 |

| **Response Profile** | | |
| --- | --- | --- |
| **Ordered Value** | **pCR** | **Total Frequency** |
| **1** | no | 45 |
| **2** | yes | 18 |

| **Probability modeled is pCR='yes'.** |
| --- |

| **Type 3 Analysis of Effects** | | | |
| --- | --- | --- | --- |
| **Effect** | **DF** | **Wald Chi-Square** | **Pr > ChiSq** |
| **Cohort** | 1 | 7.8909 | 0.0050 |
| **RACE** | 1 | 3.7908 | 0.0515 |
| **Ductal** | 1 | 2.0154 | 0.1557 |

| **Analysis of Maximum Likelihood Estimates** | | | | | | |
| --- | --- | --- | --- | --- | --- | --- |
| **Parameter** |  | **DF** | **Estimate** | **Standard Error** | **Wald Chi-Square** | **Pr > ChiSq** |
| **Intercept** |  | 1 | -4.2081 | 1.3035 | 10.4212 | 0.0012 |
| **Cohort** | **Bevacizumab** | 1 | 2.1286 | 0.7578 | 7.8909 | 0.0050 |
| **RACE** | **B** | 1 | 1.3995 | 0.7188 | 3.7908 | 0.0515 |
| **Ductal** | **yes** | 1 | 1.6815 | 1.1845 | 2.0154 | 0.1557 |

| **Odds Ratio Estimates** | | | |
| --- | --- | --- | --- |
| **Effect** | **Point Estimate** | **95% Wald Confidence Limits** | |
| **Cohort Bevacizumab vs Doxorubicin** | 8.403 | 1.903 | 37.107 |
| **RACE B vs W** | 4.053 | 0.991 | 16.582 |
| **Ductal yes vs no** | 5.374 | 0.527 | 54.764 |

| **Number of Observations Read** | 63 |
| --- | --- |
| **Number of Observations Used** | 60 |

| **Response Profile** | | |
| --- | --- | --- |
| **Ordered Value** | **pCR** | **Total Frequency** |
| **1** | no | 44 |
| **2** | yes | 16 |

| **Probability modeled is pCR='yes'.** |
| --- |

| **Type 3 Analysis of Effects** | | | |
| --- | --- | --- | --- |
| **Effect** | **DF** | **Wald Chi-Square** | **Pr > ChiSq** |
| **Cohort** | 1 | 6.7327 | 0.0095 |
| **RACE** | 1 | 5.2984 | 0.0213 |
| **Grade** | 1 | 0.0926 | 0.7609 |

| **Analysis of Maximum Likelihood Estimates** | | | | | | |
| --- | --- | --- | --- | --- | --- | --- |
| **Parameter** |  | **DF** | **Estimate** | **Standard Error** | **Wald Chi-Square** | **Pr > ChiSq** |
| **Intercept** |  | 1 | -3.8741 | 1.1899 | 10.6007 | 0.0011 |
| **Cohort** | **Bevacizumab** | 1 | 2.8755 | 1.1082 | 6.7327 | 0.0095 |
| **RACE** | **B** | 1 | 1.7229 | 0.7485 | 5.2984 | 0.0213 |
| **Grade** | **High Grade** | 1 | 0.2274 | 0.7473 | 0.0926 | 0.7609 |

| **Odds Ratio Estimates** | | | |
| --- | --- | --- | --- |
| **Effect** | **Point Estimate** | **95% Wald Confidence Limits** | |
| **Cohort Bevacizumab vs Doxorubicin** | 17.734 | 2.021 | 155.635 |
| **RACE B vs W** | 5.601 | 1.292 | 24.285 |
| **Grade High Grade vs Low Grade** | 1.255 | 0.290 | 5.431 |

| **Number of Observations Read** | 63 |
| --- | --- |
| **Number of Observations Used** | 63 |

| **Response Profile** | | |
| --- | --- | --- |
| **Ordered Value** | **pCR** | **Total Frequency** |
| **1** | no | 45 |
| **2** | yes | 18 |

| **Probability modeled is pCR='yes'.** |
| --- |

| **Type 3 Analysis of Effects** | | | |
| --- | --- | --- | --- |
| **Effect** | **DF** | **Wald Chi-Square** | **Pr > ChiSq** |
| **Cohort** | 1 | 7.8697 | 0.0050 |
| **RACE** | 1 | 6.1709 | 0.0130 |
| **Stage** | 1 | 2.9057 | 0.0883 |

| **Analysis of Maximum Likelihood Estimates** | | | | | | |
| --- | --- | --- | --- | --- | --- | --- |
| **Parameter** |  | **DF** | **Estimate** | **Standard Error** | **Wald Chi-Square** | **Pr > ChiSq** |
| **Intercept** |  | 1 | -2.5308 | 0.7118 | 12.6425 | 0.0004 |
| **Cohort** | **Bevacizumab** | 1 | 2.2954 | 0.8183 | 7.8697 | 0.0050 |
| **RACE** | **B** | 1 | 1.7263 | 0.6949 | 6.1709 | 0.0130 |
| **Stage** | **IIIA/B/C** | 1 | -1.2974 | 0.7611 | 2.9057 | 0.0883 |

| **Odds Ratio Estimates** | | | |
| --- | --- | --- | --- |
| **Effect** | **Point Estimate** | **95% Wald Confidence Limits** | |
| **Cohort Bevacizumab vs Doxorubicin** | 9.929 | 1.997 | 49.363 |
| **RACE B vs W** | 5.620 | 1.439 | 21.941 |
| **Stage IIIA/B/C vs IIA/B** | 0.273 | 0.061 | 1.215 |

| **Number of Observations Read** | 63 |
| --- | --- |
| **Number of Observations Used** | 63 |

| **Response Profile** | | |
| --- | --- | --- |
| **Ordered Value** | **pCR** | **Total Frequency** |
| **1** | no | 45 |
| **2** | yes | 18 |

| **Probability modeled is pCR='yes'.** |
| --- |

| **Type 3 Analysis of Effects** | | | |
| --- | --- | --- | --- |
| **Effect** | **DF** | **Wald Chi-Square** | **Pr > ChiSq** |
| **Cohort** | 1 | 5.2296 | 0.0222 |
| **RACE** | 1 | 6.3163 | 0.0120 |
| **ER_PR** | 1 | 2.8351 | 0.0922 |

| **Analysis of Maximum Likelihood Estimates** | | | | | | |
| --- | --- | --- | --- | --- | --- | --- |
| **Parameter** |  | **DF** | **Estimate** | **Standard Error** | **Wald Chi-Square** | **Pr > ChiSq** |
| **Intercept** |  | 1 | -3.1650 | 0.8227 | 14.7999 | 0.0001 |
| **Cohort** | **Bevacizumab** | 1 | 1.7579 | 0.7687 | 5.2296 | 0.0222 |
| **RACE** | **B** | 1 | 1.7330 | 0.6896 | 6.3163 | 0.0120 |
| **ER_PR** | **Both Neg** | 1 | 1.1235 | 0.6672 | 2.8351 | 0.0922 |

| **Odds Ratio Estimates** | | | |
| --- | --- | --- | --- |
| **Effect** | **Point Estimate** | **95% Wald Confidence Limits** | |
| **Cohort Bevacizumab vs Doxorubicin** | 5.801 | 1.286 | 26.170 |
| **RACE B vs W** | 5.658 | 1.464 | 21.857 |
| **ER_PR Both Neg vs Any Pos** | 3.075 | 0.832 | 11.373 |

| **Number of Observations Read** | 63 |
| --- | --- |
| **Number of Observations Used** | 63 |

| **Response Profile** | | |
| --- | --- | --- |
| **Ordered Value** | **pCR** | **Total Frequency** |
| **1** | no | 45 |
| **2** | yes | 18 |

| **Probability modeled is pCR='yes'.** |
| --- |

| **Type 3 Analysis of Effects** | | | |
| --- | --- | --- | --- |
| **Effect** | **DF** | **Wald Chi-Square** | **Pr > ChiSq** |
| **Cohort** | 1 | 5.8996 | 0.0151 |
| **RACE** | 1 | 6.7824 | 0.0092 |
| **HER_2_NEU** | 1 | 0.0722 | 0.7881 |

| **Analysis of Maximum Likelihood Estimates** | | | | | | |
| --- | --- | --- | --- | --- | --- | --- |
| **Parameter** |  | **DF** | **Estimate** | **Standard Error** | **Wald Chi-Square** | **Pr > ChiSq** |
| **Intercept** |  | 1 | -2.8917 | 1.0677 | 7.3347 | 0.0068 |
| **Cohort** | **Bevacizumab** | 1 | 1.8396 | 0.7574 | 5.8996 | 0.0151 |
| **RACE** | **B** | 1 | 1.7647 | 0.6776 | 6.7824 | 0.0092 |
| **HER_2_NEU** | **Neg** | 1 | 0.2344 | 0.8721 | 0.0722 | 0.7881 |

| **Odds Ratio Estimates** | | | |
| --- | --- | --- | --- |
| **Effect** | **Point Estimate** | **95% Wald Confidence Limits** | |
| **Cohort Bevacizumab vs Doxorubicin** | 6.294 | 1.426 | 27.770 |
| **RACE B vs W** | 5.840 | 1.547 | 22.039 |
| **HER_2_NEU Neg vs Pos** | 1.264 | 0.229 | 6.985 |

| **Number of Observations Read** | 63 |
| --- | --- |
| **Number of Observations Used** | 63 |

| **Response Profile** | | |
| --- | --- | --- |
| **Ordered Value** | **pCR** | **Total Frequency** |
| **1** | no | 45 |
| **2** | yes | 18 |

| **Probability modeled is pCR='yes'.** |
| --- |

| **Type 3 Analysis of Effects** | | | |
| --- | --- | --- | --- |
| **Effect** | **DF** | **Wald Chi-Square** | **Pr > ChiSq** |
| **Cohort** | 1 | 5.8018 | 0.0160 |
| **RACE** | 1 | 5.8561 | 0.0155 |
| **Triple_Negative** | 1 | 1.0205 | 0.3124 |

| **Analysis of Maximum Likelihood Estimates** | | | | | | |
| --- | --- | --- | --- | --- | --- | --- |
| **Parameter** |  | **DF** | **Estimate** | **Standard Error** | **Wald Chi-Square** | **Pr > ChiSq** |
| **Intercept** |  | 1 | -2.9071 | 0.7594 | 14.6551 | 0.0001 |
| **Cohort** | **Bevacizumab** | 1 | 1.8090 | 0.7510 | 5.8018 | 0.0160 |
| **RACE** | **B** | 1 | 1.6578 | 0.6850 | 5.8561 | 0.0155 |
| **Triple_Negative** | **yes** | 1 | 0.6792 | 0.6724 | 1.0205 | 0.3124 |

| **Odds Ratio Estimates** | | | |
| --- | --- | --- | --- |
| **Effect** | **Point Estimate** | **95% Wald Confidence Limits** | |
| **Cohort Bevacizumab vs Doxorubicin** | 6.105 | 1.401 | 26.604 |
| **RACE B vs W** | 5.248 | 1.370 | 20.094 |
| **Triple_Negative yes vs no** | 1.972 | 0.528 | 7.367 |
